# Draft Genome Sequence of the Novel, Moderately Thermophilic, Iron- and Sulfur-Oxidizing Firmicute Strain Y002, Isolated from an Extremely Acidic Geothermal Environment

**DOI:** 10.1128/mra.00149-22

**Published:** 2022-05-16

**Authors:** José Bitencourt, Larissa Scholte, Renato Oliveira, Daniel Morais, Gisele Nunes, Ivan Nancucheo, Rafael Valadares, David S. Holmes, D. Barrie Johnson, Ronnie Alves, Guilherme Oliveira

**Affiliations:** a Instituto Tecnológico Vale, Belém, Pará, Brazil; b René Rachou Institute, Fiocruz, Belo Horizonte, Minas Gerais, Brazil; c Luiz de Queiroz College of Agriculture, University of São Paulo—ESALQ-USP, Piracicaba, Sao Paulo, Brazil; d Facultad de Ingeniería y Tecnología, San Sebastian University, Concepción, Chile; e Center for Bioinformatics and Genome Biology, Centro Ciencia & Vida, Fundación Ciencia & Vida and Facultad de Medicina y Ciencia, San Sebastian University, Santiago, Chile; f School of Natural Sciences, Bangor University, Bangor, United Kingdom; g Pós-Graduação em Ciência da Computação (PPGCC), Universidade Federal do Pará, Belém, Brazil; University of Southern California

## Abstract

We report the draft genome sequence of the Firmicute strain Y002, a facultatively anaerobic, acidophilic bacterium that catalyzes the dissimilatory oxidation of iron and sulfur and the reduction of ferric iron. Analysis of the genome (2.9 Mb; G+C content, 46 mol%) provided insights into its ability to grow in extremely acidic geothermal environments.

## ANNOUNCEMENT

We report the draft genome sequence of the Firmicute strain Y002, a moderately thermophilic, extremely acidophilic, facultative anaerobe isolated from an acidic (pH 3.3) geothermal (78°C) site within Yellowstone National Park (WY, USA) (1, 2).

Firmicute strain Y002 was grown in a liquid medium containing 20 mM ferrous iron and 0.05% (wt/vol) yeast extract (pH 1.7) at 45°C; the biomass was harvested by centrifugation and the DNA extracted using the FastDNA Spin kit for soil (2). Following the manufacturer’s recommendations, whole-genome sequencing was carried out using the Illumina MiSeq platform. Two paired-end libraries were produced using the Nextera DNA sample preparation kit, generating 1,627,254 paired-end reads with coverages of 159× and 93× for the libraries. The reads were trimmed and filtered using FastX-Toolkit v0.0.13 (http://hannonlab.cshl.edu/fastx_toolkit/) (Phred score, 20; minimum read length, 20; minimum percentage of high-quality bases, 80%) (3), generating 1,585,528 high-quality paired-end reads. *De novo* assembly was carried out using SPAdes v3.7 (4), resulting in 135 contigs comprising a single chromosome of 2.9 Mb, with a G+C content of 46% and an assembly coverage of 223×. The final assemblies were annotated using the Prokaryotic Genome Annotation Pipeline v3.3, with the best-placed reference protein set and GeneMarkS+ as the annotation methods (5). The draft genome comprises 2,873,735 bp with an *N*_50_ value of 95,731 bp. Using CheckM v1.1.2 (6), a completeness of 98.08% was predicted, with 1.79% contamination. The genome potentially encodes 2,864 protein-coding genes (of which 1,291 have predicted functions), 46 tRNA genes, and 6 rRNA genes, consisting of 2 each 16S rRNA, 23S rRNA, and 5S rRNA genes. Unless otherwise specified, default parameters were used for the software analysis.

The Firmicute strain Y002 has an absolute requirement for an organic carbon source and cannot fix carbon dioxide, even in CO_2_-enriched atmospheres (2). Interestingly, the genome was found to include three gene clusters predicted to encode proteins involved in carbon assimilation via the Calvin-Benson-Bassham (CBB) cycle, including ribulose-1,5-bisphosphate carboxylase/oxygenase (RubisCO) and the LysR-type transcriptional regulator CbbR (7). However, the genome lacks genes encoding carboxysomes, which are typically associated with CBB genes (8).

The genome was predicted to encode *sor*, supporting the previous report (2) that this bacterium can catalyze the dissimilatory oxidation of S^0^. The uptake of sulfate and oxidation of sulfide appear to be mediated via *sqr* (*sat* and *cys*CP) (9). Its diversified metabolism of sulfur compounds could help it to exploit high-temperature biomining environments, in addition to geothermal sites.

Genome interrogation suggests that iron acquisition and homeostasis are mediated through the production of siderophores, transcriptional regulators, several Fe-S binding proteins, and *hem*N, *nif*U, *efe*U, and *yfe*B (10, 11). The genome is also predicted to have a copy of *feo*E, which is important for survival during anaerobic iron respiration (12), and an Nramp family of proteins that are involved in cellular responses to fluctuating environmental supplies of metal ions (13). Experimental data are required to confirm these predictions.

In summary, analysis of the Firmicute strain Y002 genome has provided preliminary insight into the distinctive characteristics that facilitate its growth in low-pH thermal environments, suggesting possible uses in commercial bioleaching operations.

### Data availability.

The whole-genome shotgun project was deposited at DDBJ/EMBL/GenBank under accession number MPDK01000000; the assembly and raw data can be found under SRA accession numbers SRR18070407 and SRR18070406. The version described here is version MPDK00000000.1.
